# An automated pipeline for the screening of diverse monoterpene synthase libraries

**DOI:** 10.1038/s41598-019-48452-2

**Published:** 2019-08-15

**Authors:** Nicole G. H. Leferink, Mark S. Dunstan, Katherine A. Hollywood, Neil Swainston, Andrew Currin, Adrian J. Jervis, Eriko Takano, Nigel S. Scrutton

**Affiliations:** Manchester Synthetic Biology Research Centre for Fine and Speciality Chemicals (SYNBIOCHEM), Manchester Institute of Biotechnology and School of Chemistry, University of Manchester, Manchester, United Kingdom

**Keywords:** Enzymes, Metabolic engineering

## Abstract

Monoterpenoids are a structurally diverse group of natural products with applications as pharmaceuticals, flavourings, fragrances, pesticides, and biofuels. Recent advances in synthetic biology offer new routes to this chemical diversity through the introduction of heterologous isoprenoid production pathways into engineered microorganisms. Due to the nature of the branched reaction mechanism, monoterpene synthases often produce multiple products when expressed in monoterpenoid production platforms. Rational engineering of terpene synthases is challenging due to a lack of correlation between protein sequence and cyclisation reaction catalysed. Directed evolution offers an attractive alternative protein engineering strategy as limited prior sequence-function knowledge is required. However, directed evolution of terpene synthases is hampered by the lack of a convenient high-throughput screening assay for the detection of multiple volatile terpene products. Here we applied an automated pipeline for the screening of diverse monoterpene synthase libraries, employing robotic liquid handling platforms coupled to GC-MS, and automated data extraction. We used the pipeline to screen pinene synthase variant libraries, with mutations in three areas of plasticity, capable of producing multiple monoterpene products. We successfully identified variants with altered product profiles and demonstrated good agreement between the results of the automated screen and traditional shake-flask cultures. In addition, useful insights into the cyclisation reaction catalysed by pinene synthase were obtained, including the identification of positions with the highest level of plasticity, and the significance of region 2 in carbocation cyclisation. The results obtained will aid the prediction and design of novel terpene synthase activities towards clean monoterpenoid products.

## Introduction

Terpenoids are a large class of natural products with more than 80,000 compounds identified up to now (http://dnp.chemnetbase.com). Most terpenoids are found in plants with varying biological roles in, for example, inter-species communication, intracellular signalling, and defence against predators^1^. All terpenoids originate from the linear C5 isoprenoid precursor substrates isopentenyl diphosphate (IPP) and dimethylallyl diphosphate (DMAPP). Prenyl transferases join the IPP and DMAPP molecules to form prenyl diphosphate substrates of different lengths (C10, C15, C20, etc.), which are used by terpene cyclases or synthases to produce structurally complex linear and cyclic mono-, sesqui-, di-, or larger terpene scaffolds. Due to their structural diversity, terpenoids have a wide range of industrial applications as, for example, precursors for pharmaceuticals, flavourings, fragrances, antiseptics, pesticides, and other household products, as well as alternative fuels and bioplastics^2–4^.

**Figure 1:**
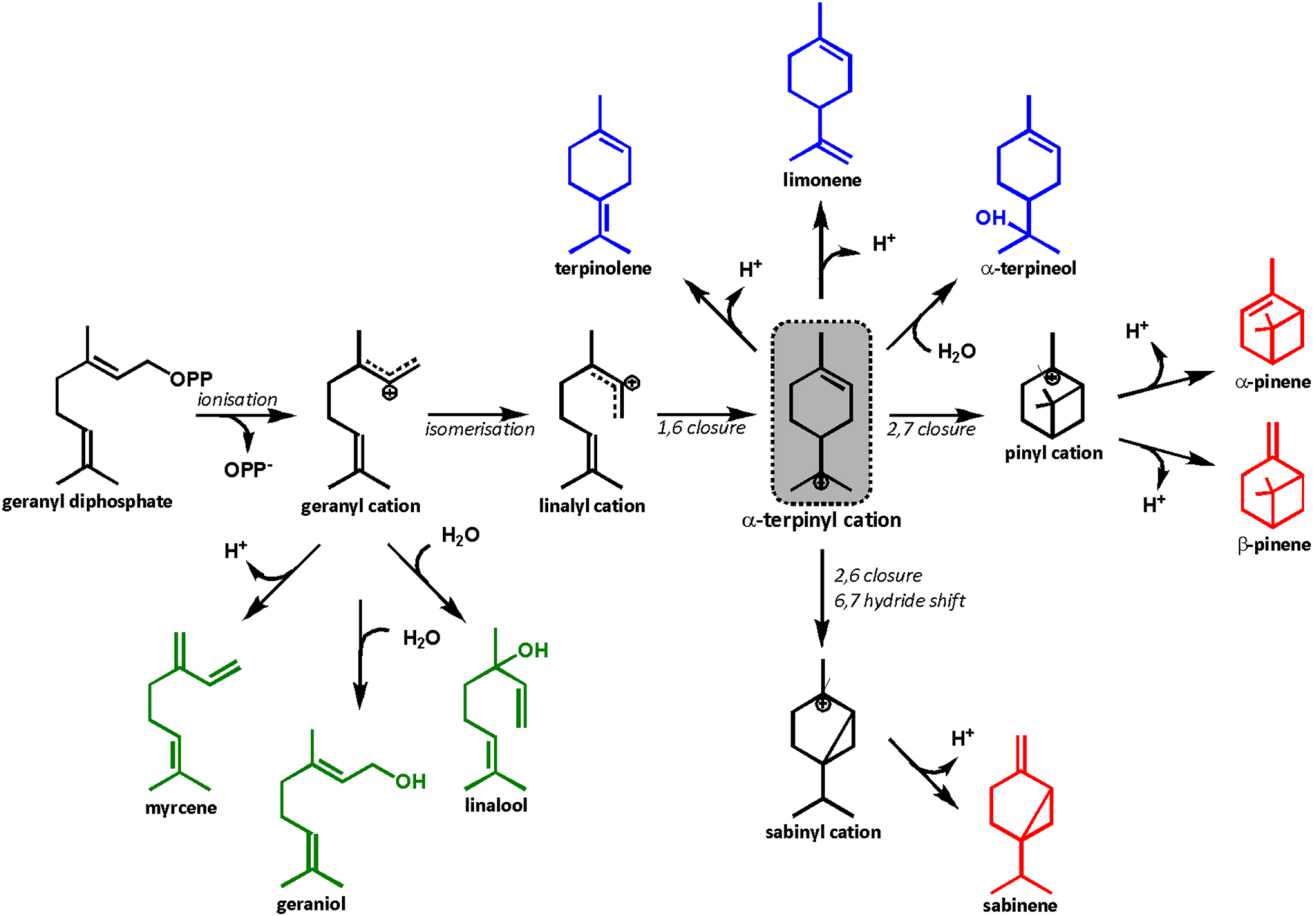
Proposed cyclisation cascade catalysed by (-)-α-pinene synthase from *Pinus taeda* (PinS)^28,39^. The reaction starts with the ionisation of the geranyl diphosphate substrate (GPP), yielding the geranyl cation. The carbocation can undergo a range of cyclisations and hydride shifts before the reaction is terminated by deprotonation or water capture. Linear products are highlighted in green, monocyclic products in blue, and bicyclic products are shown in purple. The product mixture produced by PinS consists of: α-pinene (84%), β-pinene (9%), geraniol (3%), myrcene (2%), and limonene (1%).

Previously we created a flexible production platform for the generation of diverse monoterpene hydrocarbon scaffolds via expression of various plant monoterpene cyclases/synthases (mTC/S) into an engineered *E*. *coli* strain containing a hybrid yeast-bacterial heterologous mevalonate (MVA) pathway^5,6^. Most mTC/S enzymes expressed in the engineered strain produced a mixture of monoterpene products; however the generation of pure products is desirable for most commercial applications. Multiple products may arise due to the highly branched cyclisation reactions catalysed by mTC/S enzymes involving unstable carbocation intermediates (Fig. 1). All mTC/S catalysed reactions are initiated with the metal-dependent ionisation of GPP resulting in the geranyl cation, the first reactive carbocation intermediate. After the initial ionisation step, each mTC/S enzyme effectively provides little more than a scaffold for the cyclisation cascade which directs the conformation of the carbocation intermediates. The relatively inert active sites of mTC/S enzymes show a high degree of functional plasticity, which is the capacity to undergo change via a small number of mutations, resulting in product diversification^7–10^. As a result, rational structure-guided engineering of mTC/S enzymes is challenging due to the lack of a clear structure-function relation between the enzyme active site and the cyclisation cascade catalysed. In addition, despite the description of numerous mTC/S enzymes in the literature, only a handful of crystal structures are available to date^11–17^. However, even in the presence of detailed structural information, the challenge remains to pinpoint individual amino acid contributions to the cyclisation cascade, and therefore ultimately product outcome.

Proteins can be engineered with little or no prior knowledge of the structure/function relationship by screening for improved variants in a process called directed evolution^18^. The development of high-throughput (HTP) screening assays for terpene synthase activity is particularly challenging due to the fact that multiple products may be produced from a single substrate. Examples of HTP terpene synthase assays include a colorimetric assay based on pyrophosphate release^19^, and a screen for cyclisation activity using a modified substrate that detects an artificial cyclisation by-product in a coupled enzymatic reaction^20^. Other assays allow the detection of enhanced terpene production *in vivo* where terpene synthases compete for the available GPP pool with a heterologous carotenoid biosynthesis pathway^21,22^, or include a fluorescent-based genetically-encoded biosensor for enhanced intracellular isoprene production^23^. However, none of these examples allows the rapid detection of multiple volatile terpene compounds in a high-throughput fashion.

**Figure 2:**
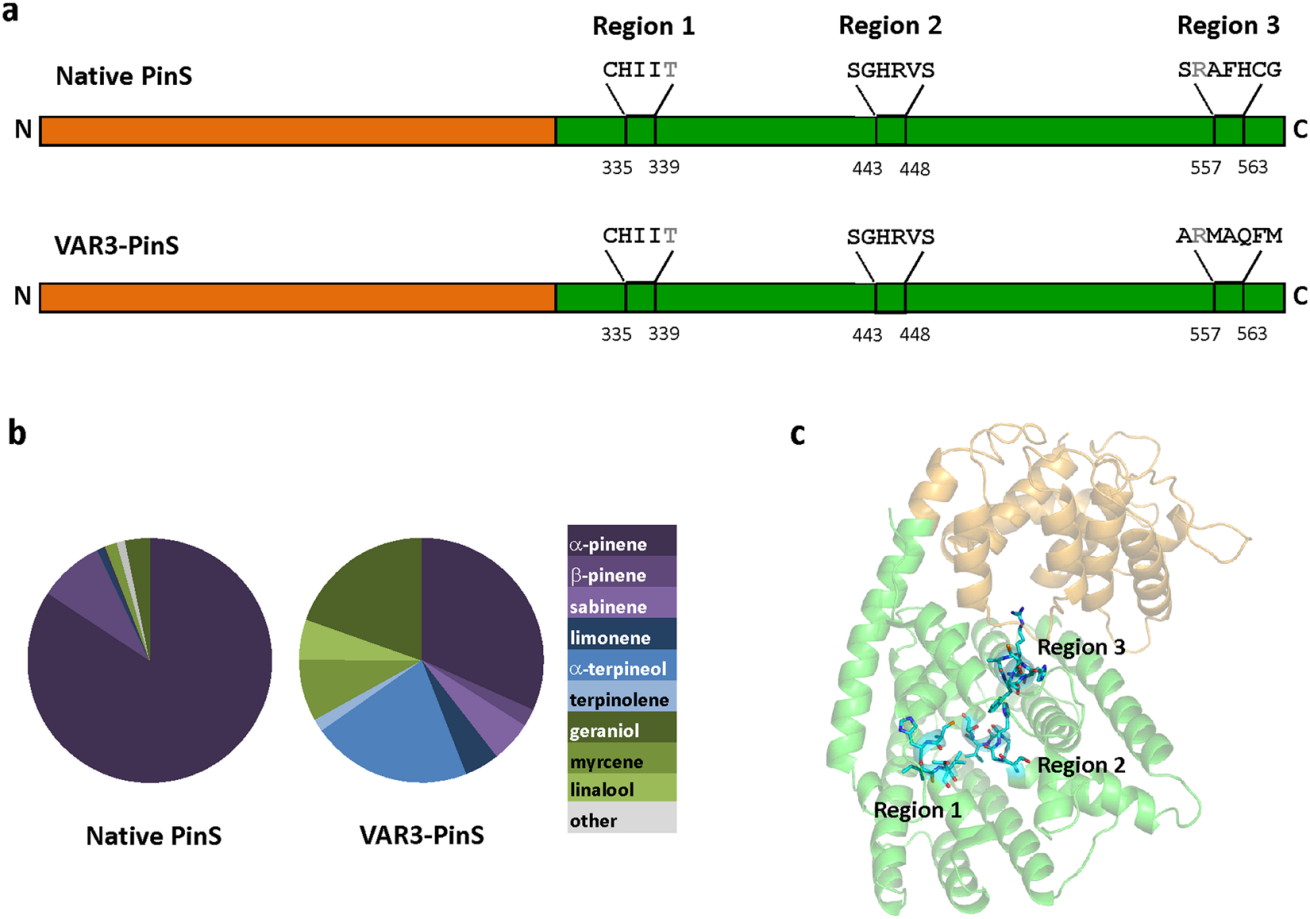
PinS domain organisation and location of the targeted amino acids. (**a**) Domain organisation of native PinS from *Pinus Taeda* (Uniprot ID: Q84KL6) and VAR3-PinS with indicated plasticity regions targeted in this study. The N-terminal domain of unknown function is shown in orange and the C-terminal class I terpene cyclase domain is shown in green. The amino acid residues in the three plasticity regions targeted in this study are indicated. Residues at positions 339 and 558 (in grey) were not targeted as they are highly conserved among plant mTC/S^8^. (**b**) Relative product profiles of native PinS and VAR3-PinS (data obtained from Leferink *et al*.^8^), the latter was used as template for further mutagenesis in this study. (**c**) The three targeted areas mapped onto a homology model of native PinS (SWISS-MODEL, *Abies grandis* α-bisabolene synthase (PDB: 3SAE^40^) was used as a template, which has 44.8% sequence identity with (-)-α-pinene synthase from *Pinus taeda*). The N-terminal domain is shown in orange, the C-terminal terpene cyclase domain in green, and the three targeted plasticity regions are highlighted in cyan sticks. The amino-acid sequences of recombinant native and VAR3-PinS are shown in Table S1 in the Supplementary Information available.

Here we applied a monoterpenoid screening pipeline which employs a GC-MS based automated pipeline utilising a liquid handling robotic platform for the HTP screening of mTC/S variant libraries *in vivo*^24^. We targeted 16 amino acid residues that are part of three previously identified conserved plasticity regions^8^ in a (-)-α-pinene synthase variant (VAR3-PinS) that produces an equal mixture of linear, monocyclic and bicyclic monoterpenoid products (Fig. 2). To address the numbers problem in directed evolution^25^, the NBT degenerate codon (12 codons, 11 amino acid residues) was selected for introduction at the 16 target positions. The mostly polar and hydrophobic residues encoded by NBT correspond to the residues naturally found in the plasticity regions of the plant mTC/S enzyme family^8^, which limits the search to the parts of the sequence space that encode functional variants and avoid areas of non-functional variants. Using the pipeline, approximately 1000 colonies were screened, and over 65 unique sequences with associated product profiles were detected. There was good agreement between the results obtained from the screening pipeline and results obtained from subsequent conventional shake-flask cultures. The positions with the highest level of plasticity, where a change in amino acid composition resulted in altered product profiles, are position 335 in region 1, and positions 557, 559, 560 and 561 in region 3. The variant product profiles provide useful insights into the PinS catalysed reaction, where residues in region 2 are shown to be essential for carbocation cyclisation. The data obtained in this study provide a better understanding of the molecular determinants for product outcome in PinS, and the pipeline can be directly applied to other (mono)terpene synthase libraries.

## Results and Discussion

### Detection of multiple products using an automated HTP monoterpenoid screening pipeline

**Figure 3:**
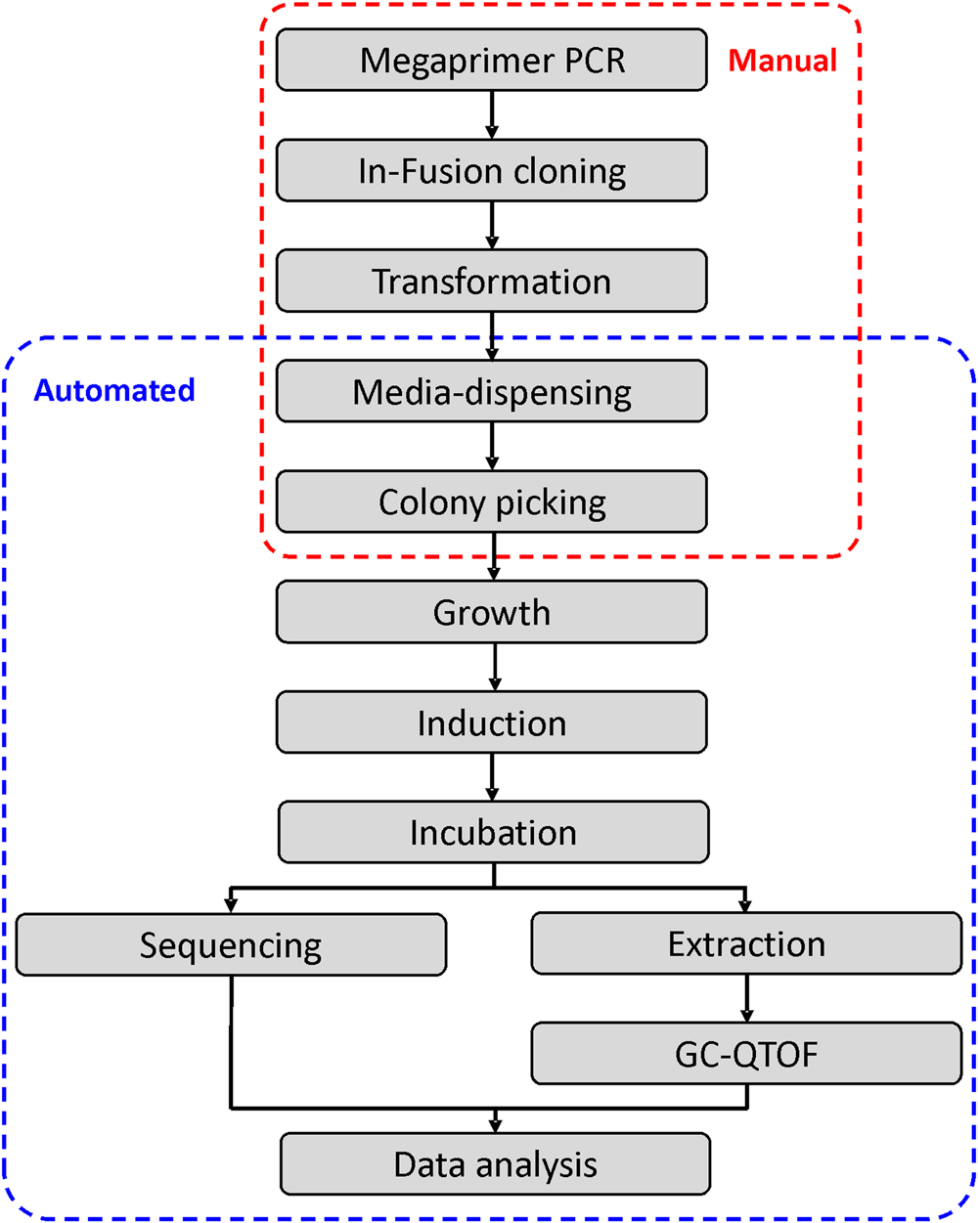
Schematic overview of the *in vivo* monoterpenoid screening pipeline in *E*. *coli*. The pipeline is designed for the use of 96-well plates to ensure compatibility with robotic liquid handling platforms for easy sample processing. Manual and automated steps are indicated with red and blue dashed boxes, respectively. The media-dispensing and colony picking steps can be performed either manually or automated. Typically, three 96-well plates (288 samples) can be processed per day^24^.

The automated monoterpenoid screening pipeline used in this study builds on an established pipeline developed previously for the screening of variants of a limonene production pathway *in vivo* in engineered *E*. *coli* towards improved titres^24^. Established growth conditions and robotic liquid handling steps for sample preparation were employed, while the automated data extraction scripts were adapted for the detection of additional monoterpene compounds, allowing for the screening of variant product profiles. Briefly, standard two-phase shake-flask culture conditions were scaled down to a 96-deepwell plate format, allowing the growth of 96 individual two-phase cultures with organic overlay (dodecane) for product extraction. Pathway induction, organic phase extraction, and sample processing for GC-MS analysis, were performed using a robotic liquid handling platform. The pipeline was designed to be directly compatible with rapid analysis using a GC-QTOF Mass Spectrometer equipped with a 96-well plate auto-sampler. Data analysis was performed with automated data extraction scripts^24,26^. In addition, the pipeline allowed for parallel 96-well plate format automated sequencing services and data analysis (Fig. 3). To determine if the pipeline is suitable to screen mTC/S product profiles in HTP, we compared the product profiles obtained using the pipeline to those previously obtained in conventional shake-flask cultures for native (-)-α-pinene synthase (PinS) and a PinS variant that produces a mixture of monoterpenoid products (VAR3-PinS, Fig. 2b). Both enzymes were introduced into our dual-plasmid monoterpenoid production platform, which consists of a previously described IPTG-inducible heterologous MVA pathway^6^ encoded on the first plasmid (pMVA), and a tetracycline inducible truncated GPP synthase (trGPPS) followed by the native PinS or VAR3-PinS gene encoded on the second plasmid (See Fig. S1 in the Supplementary Information). Samples were processed and analysed using the automated screening pipeline described above. Initially, we attempted a targeted analysis of 9 different monoterpene compounds produced by native PinS and VAR3-PinS: α-pinene, β-pinene, sabinene, β-myrcene, limonene, terpinolene, linalool, α-terpineol, and geraniol. However, due to limited organic solvent compatibility with robotic liquid handling, the monoterpenes terpinolene, linalool, α-terpineol, and geraniol were masked by the dodecane solvent peak during GC-QTOF analysis (See Fig. S2 in the Supplementary Information). During the robotic liquid handling steps, involving pressure sensing of liquid detection, the 96-wells plates are exposed, hence only solvents with relatively low volatility and low viscosity can be used. Even though dodecane co-elutes with some of the target compounds, other solvents such as nonane or larger alkanes are not compatible with the pipeline due to increased volatility or viscosity respectively. In addition, the data extraction scripts cannot distinguish between β-myrcene and sabinene as they have very similar retention times and mass spectra when measured using the GC-MS method compatible with the pipeline (See Fig. S2 and Table S5 in the Supplementary Information). Therefore, the initial screen was limited to 4 relevant monoterpenes peaks: α- and β-pinene, limonene and the combined sabinene/β-myrcene peak. The relative product profiles obtained for native PinS and VAR3-PinS using the automated pipeline is in good agreement with the relative product profiles previously obtained in shake-flask cultures (Fig. S3 in the Supplementary Information), suggesting that the pipeline is suitable for HTP screening of mTC/S variant libraries.

### Screening of a pinene synthase library for variants with altered product profiles

Previously, we have shown that amino acids affecting product outcome are clustered and located at conserved positions around the enzyme active site within the plant mTC/S enzyme family^8^. A few mutations in these regions of high-plasticity can result in drastically altered product outcomes. We applied the automated pipeline for the screening of a VAR3-PinS library with further mutations in three plasticity regions with the aim to generate additional sequence-function data for the better understanding of the molecular determinants for cyclisation in PinS. Focussed libraries were created in which 16 amino acid positions, located in three plasticity regions, were diversified using the NBT degenerate codon. This unusual degenerate codon covers mostly polar, and hydrophobic residues, and includes codons encoding Gly, Ala, Val, Leu, Ile, Phe, Pro, Cys, Thr, Ser (2x) and Arg residues (See Table S2 in the Supplementary Information). The NBT codon purposely excludes charged residues, except Arg which is present as a result of the nature of the codon table, thereby mimicking amino acid occurrence in the identified areas of plasticity in plant mTC/S homologues found in nature^8^. The libraries containing the NBT codon will therefore likely contain a large number of active variants. Variant libraries were introduced into the dual-plasmid monoterpene production platform, which allows for the efficient mutagenesis and cloning of variant genes on the smaller gene module only. The Megaprimer PCR method^27^ was used for the targeted introduction of mutations, followed by scar-less cloning using the In-Fusion cloning method.

**Figure 4:**
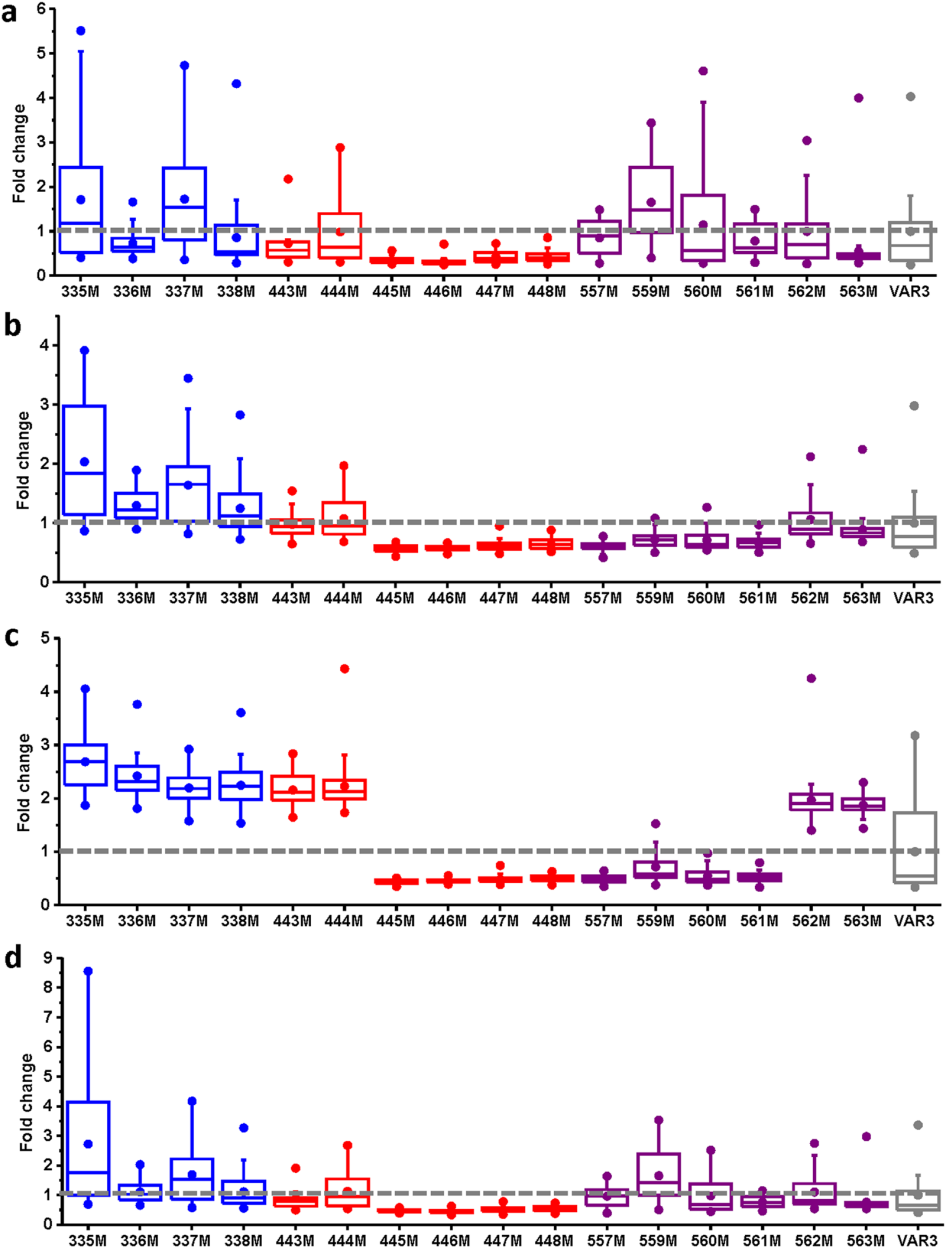
Screening and analysis of the VAR3-PinS libraries for diverse monoterpene production. A targeted analysis was conducted for the monoterpene products α-pinene (**a**), β-pinene (**b**), limonene (**c**) and sabinene/myrcene (**d**). Libraries with mutations in region 1 are in blue, region 2 in red, and region 3 in purple. The median fold-change over PinS-VAR3 (in grey) and the upper and lower quartiles are indicated. The data plotted for VAR3-PinS represents the fold change of each individual data point compared to the median of all data-points obtained for VAR3-PinS. The number of data-points for each library is between 35 and 40.

In total, 16 libraries, each containing up to 12 variant codons, were created using plasmid pBb-PinS_var3 as a template (See Table S4 in the Supplementary Information) and co-transformed with pMVA into *E*. *coli* DH5α cells. Using the automated pipeline, at least 40 colonies were screened for each library (the probability that each variant is represented, p > 0.95), and a targeted analysis for the production of α-pinene, β-pinene, limonene and myrcene/sabinene was performed. Figure 4 shows the fold-change observed in each library for all four monoterpene peaks analysed. These initial screening results show a large variation in the fold-change over VAR3-PinS between the various libraries. For example, all colonies screened for the libraries at positions 445, 446, 447 and 448 produce less than the VAR3-PinS parent enzyme for all 4 peaks analysed (fold-change < 1.0), which is indicative of a large number of variants with low or no activity in these libraries. In contrast, the positions that show the greatest plasticity result in libraries that contain a wide range of titres (fold-change > 3.0) for multiple monoterpene products, for example the libraries created at positions 335, 337 and 559. It should be noted that the significant variation observed in peak intensities for VAR3-PinS, most notably for limonene (Fig. 4, panel C), is most likely due to some variations in growth and induction conditions, as VAR3-PinS was present as a control on each 96 well plate screened. Such variations in growth and induction conditions are unlikely the cause of variation in fold change observed within each library, as each library was contained on a single 96 well plate, and therefore are the result of changes in terpene synthase activity.

Even though the targeted analysis only allowed the detection of a limited number of monoterpenoid compounds, the screening pipeline was effective at identifying active variants with alternative product profiles, and is therefore suitable as an initial HTP screen to enrich the variant pool with active variants. The pipeline can be easily adapted for the detection of other (mono)terpenoids by modifying the data extraction scripts, including untargeted metabolomics analysis if the compounds produced are unknown^26^.

### Validation of the automated pipeline in shake-flask cultures

**Figure 5:**
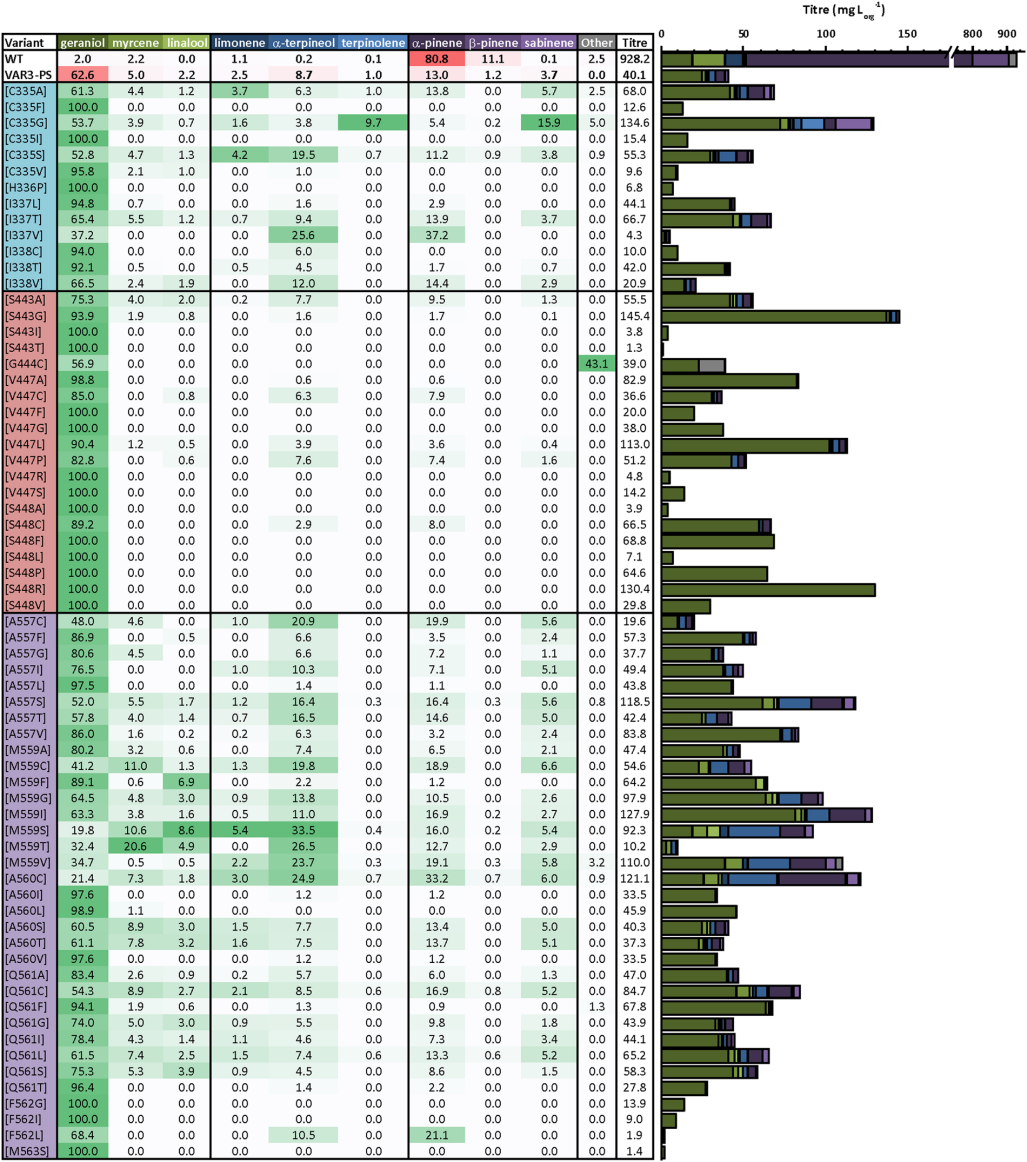
Relative product profiles obtained for each active variant using VAR3-PinS as template. A comparison of the relative titres of the 9 common monoterpene products found in native and VAR3-PinS is shown. A darker shade of green indicates a greater relative accumulation of that compound. Variants with mutations in region 1 are in blue, region 2 in red, and region 3 in purple. Data for native (WT) and VAR3-PinS was obtained from Leferink *et al*.^8^. Other monoterpenoid products detected are β-phellandrene (for variants C335A, C335G, A557S and M559V) and sabinene-hydrate (for variants C335A, C335G, and A560C). Other sesquiterpenoid products detected are farnesene (for variants M559V and Q561F), nerolidol (for variant M559V), and farnesol (for variant G444C). A full breakdown of the product profiles can be found in Table S6 in the Supplementary Information.

From the enriched pool of variants from the initial screen we selected approximately 350 ‘active’ variants, that each produce at least one of the target compounds above background level (See Experimental Section), for sequencing analysis, which resulted in the identification of 94 unique variants, each containing a single amino-acid mutation. Glycerol stocks of these 94 clones were re-streaked on fresh agar plates, followed by colony screening in conventional two-phase shake-flask cultures, which resulted in 67 unique PinS variant sequences with associated detailed product profiles (Fig. 5). For the additional 27 variants no monoterpene products were detected in the shake-flask cultures, and these therefore represent false-positive results from the initial screen. The 67 unique sequences correspond to 40% of active variants across the sequence space searched. The peak intensities obtained for each individual variant using the automated screening pipeline are shown in Fig. S4 of the Supplementary Information.

Overall there is a good agreement between the results of the initial screen and the results obtained from the scale-up, demonstrating that the screening pipeline is effective at identifying variants with altered product profiles. For example, the libraries containing mutations at positions 335 and 559 demonstrated a range of titres for multiple target products in the initial screen (see Figs 4 and S4 in the Supplementary Information), which is confirmed by the large number of different active variants observed for these positions (6 and 8, respectively), each producing a variety of products resulting in product profiles that are different from VAR3-PinS (Fig. 5). Generally, there is good quantitative directional agreement between the initial screen and the scale-up. Some of the variants showing the overall largest peak intensities in the initial screen (e.g. C335A, G, and S, I337T, A557S, M59C, T, and V, A560C, and F562P and L) also produced the highest titres in the shake-flask cultures, excluding geraniol, except F562P and F562L. The latter two variants did not produce any detectable monoterpenoids in the shake-flask cultures, and are thus false-positives. The increased production of α-pinene observed in several A557 and M559 variants, compared to VAR3-PinS, is also reflected in the initial screen. Interestingly, most variants with mutations in the second plasticity region (positions 443–448) that produced relatively low amounts of detectable products (i.e. less than VAR3-PinS) in the initial screen did produce significant amounts of products in the scale-up. In particular, these variants produced products that could not be accurately detected in the initial screen (e.g. α-terpineol and geraniol). To establish how different each variant product profile is to VAR3-PinS a Euclidean distance was calculated for each variant product distribution profile. A Euclidean distance of zero corresponds to an identical product distribution to VAR3-PinS, and the mutations that have the greatest effect on moving the product distribution away from VAR3-PinS have the highest scores (See Table S8 in the Supplementary Information). Many variants with mutations in plasticity region 2 have a relatively high score (>0.4) as they only produce geraniol and no other monoterpenoid products. Interestingly, several positions (e.g. 335, 559, 560 and 561) have variants amongst the highest (>0.3) as well as lowest (<0.1) scoring profiles, suggesting that different mutations have a different effect on the product outcome. However, as many variants have similar or identical Euclidean scores, not all product profiles are significantly different from VAR3-PinS or each other.

Despite some limitations, (i.e. the number of variants that can be screened due to dependence on GC-MS, obtaining exact production titres due to overall lower production levels in the 1-ml deepwell plate^24^, and the masking of certain products due to solvent overlap), the above results show that the automated pipeline developed in this study can be used as a rapid initial screen to enrich the library with variants that show distinct altered product profiles (e.g. variant product profiles and/or increased product titres) in a single measurement. It is unlikely that a large number of false negatives were discarded after the initial screen as most variants produced a mixture of compounds, and, apart from geraniol, all other peaks not visible in the initial screen (terpinolene, linalool, and α-terpineol) are mainly produced as minor products by VAR3-PinS and most other variants. The fact that the screen did pick up many variants that do produce geraniol as the main product, suggests that the threshold for the selection of ‘active variants’ is sufficiently low. As such, the pipeline is a useful addition to the already existing high-throughput terpene synthase screening methods which can only screen for an overall increase in terpene synthase (cyclisation) activity^19,20^ or enhanced terpene titres *in vivo*^21,22^.

### Rare charged residues in the plasticity regions of pinene synthase are essential for activity

Further analysis of amino acid residue composition and occurrence for each library reveals interesting insights. As only active variants detected in the automated screening pipeline were sent for sequencing analysis, not all codons were represented equally across the libraries. Codons encoding Gly and Cys residues were the most detected codons across all libraries, with a relative occurrence of 14.0 and 17.7% respectively. Codons encoding Pro and Arg were the least detected codons, with a relative occurrence of 2.2 and 0.8% respectively (See Table S7 in the Supplementary Information). This is in line with the fact that terpene synthases generally have relatively inert active sites consisting of mainly polar and non-polar residues^7,8^. These results also shed more light on the potential role of each amino acid position in the cyclisation cascade and therefore product outcome in PinS catalysed reactions.

**Figure 6:**
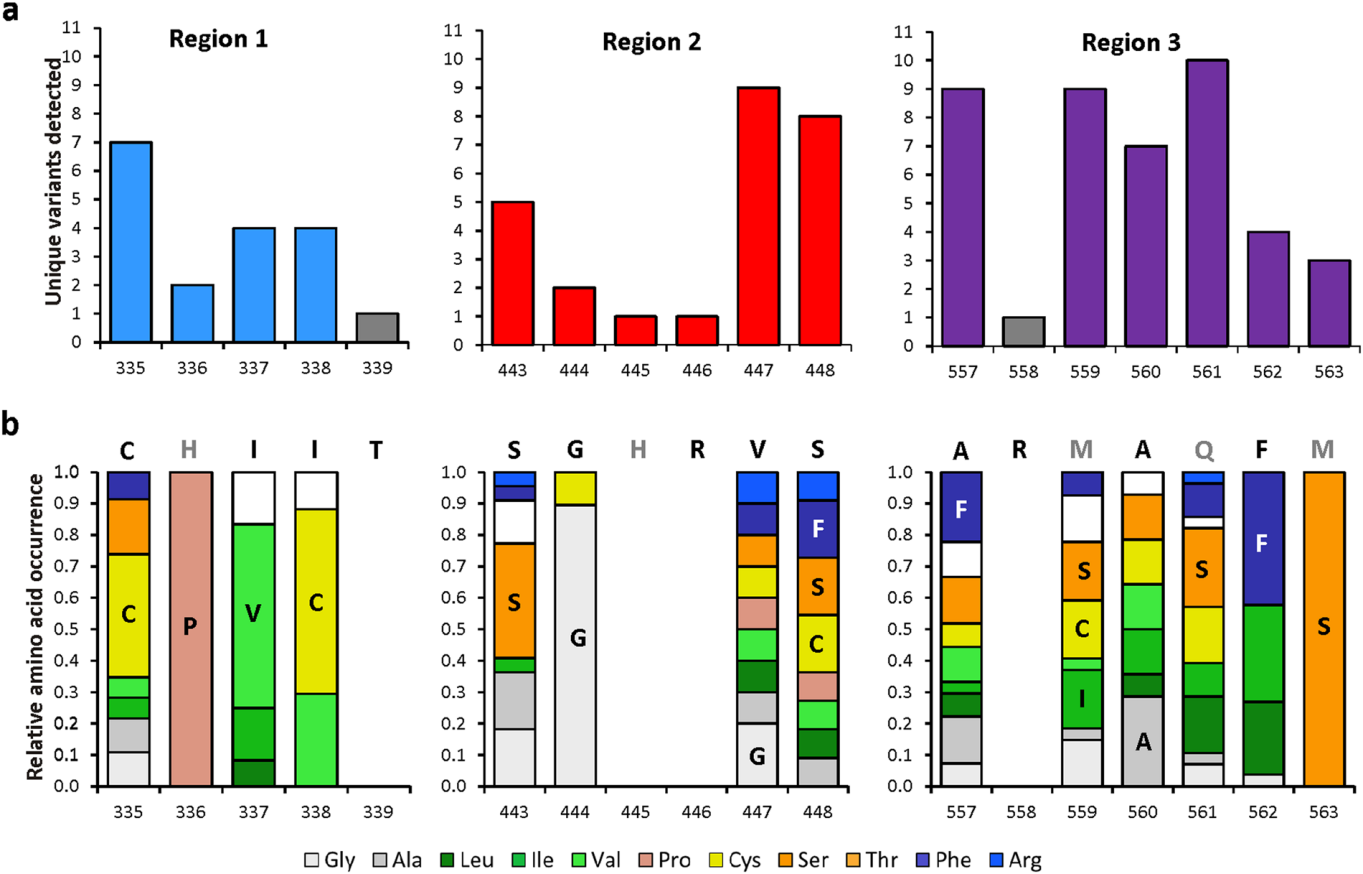
Sequence analysis of active PinS variants. (**a**) The number of unique active variants detected per mutated amino acid position, including native and VAR3-PinS residues. Bars marked in grey (positions 339 and 558) were not mutated in this study as they appeared highly conserved among plant mTC/S enzymes^8^. (**b**) Relative amino acid occurrence per mutated position for variants with confirmed activity in the shake-flask scale-up. The sequence of VAR3-PinS, the template used for mutagenesis, is shown above the bars. Residues marked in grey are not included in the NBT codon. The most occurring residue at each position is indicated in the relevant coloured bar for each position. No sequencing data is available for positions 339 and 558 (not targeted) and 445 and 446 (no active variants obtained). The data used to generate this graph is shown in Table S7 in the Supplementary Information.

A plasticity score for each given amino acid position is calculated as being the intersection between the observed amino acid distribution and a perfectly uniform distribution. If the observed distribution was perfectly uniform (i.e. if all amino acids were observed with equal frequency) the plasticity score is one. Conversely, if no mutations were observed at that position, the plasticity score is zero (See Table S9 in the Supplementary Information). Some positions have a clear ‘preference’ (>50% of variants sequenced) for a certain residue, such as positions 337, 338, 444, and 563, which all have relatively low plasticity scores. Those positions with a relatively high plasticity score (i.e. 335, 447, 448, and most positions in region 3) can accommodate a mixture of residues (Fig. 6). Those positions that show the highest level of plasticity also produce the greatest product diversity; this is particularly true for the positions in region 3. Previous mutagenesis studies on mTC/S enzymes have shown that the equivalent residues at position 335 is highly important for product outcome in a pinene synthase from *Abies grandis*^28^ (Cys372), limonene synthase (LimS) from *Mentha spicata*^8,29^ (Asn345), cineole synthase from *Salvia fruticosa*^17^ (Asn338), two sabinene hydrate synthases from *Thymus vulgaris*^30^, and a fungal cineole synthase^31^ (Asn136). Residues in region 3 have been implicated in the fine-tuning of product outcome in more complex cyclisation cascades resulting in bi-cyclic products, as we have shown previously for PinS and fenchol synthase from *Lavandula viridis*, where mutations in region 3 resulted in a mixture of products^8^. Other examples include the F597W mutation in a β-pinene synthase from *A*. *grandis*, which results in a flipped α/β-pinene ratio^28^; and the presence of a Leu or Phe at position 596 in carene and sabinene synthases from *Picea sitchensis*, which promotes the formation of carene or sabinene respectively^32^.

The involvement of highly reactive and unstable carbocations in terpene synthase catalysed cyclisation cascades, which could potentially alkylate and inactive the enzyme, typically results in relatively inert active sites containing mostly nonpolar residues^7^. Positively charged residues (His and Arg) are extremely rare in the identified plasticity regions within the plant mTC/S family, and they are essentially only observed in regions 1 and 2 of bi-cyclic mTC/S enzymes from pine trees, where they show a high degree of conservation^8^. One notable exception is the highly conserved Arg residue from region 3, which is found in all plant mTC/S so far (equivalent to Arg558 in PinS). Previously, the accumulation of S443I, H445G, R446P and S448I mutations in region 2 of PinS resulted in an inactive enzyme variant^8^. The results obtained in this study show that any mutation of H445 and/or R446 results in inactive enzyme variants, implying that these rare charged residues are essential for PinS activity. Equally, the His336 residue in region 1 also seems essential for activity, as only a single alternative variant was detected in our screen (H336P), but this variant is essentially inactive as it only produces very low amounts of geraniol, which may be attributed to endogenous *E*. *coli* activity^5,33^. Small amounts of geraniol and farnesol are produced as a result of endogenous *E*. *coli* activity. Previously, we have demonstrated that in the absence of a terpene synthase, *E*. *coli* cells harbouring the endogenous MVA pathway produce 5–10 mg/L_org_ of geraniol^5^, which means that the amount of geraniol produced in all variants is likely overestimated, especially for variants with a relative low titre. Positions 443, 447 and 448 in region 2 can accommodate a variety of residues resulting in active enzyme variants. Interestingly, all variants with mutations at these positions in region 2 mainly produce linear products (mostly geraniol, but also β-myrcene and linalool), suggesting an important role in isomerisation and/or cyclisation for plasticity region 2 in PinS. Additionally, the equivalent region in LimS was also found to be important in the cyclisation reaction, however in LimS mutations in region 2 resulted in more complex (bi-cyclic) reaction products^8^.

## Conclusions

Here we have applied an automated pipeline for the screening of diverse monoterpene synthase libraries and demonstrated that the pipeline was effective at identifying variants with altered product profiles, which were confirmed when the production was scaled up to traditional two-phase shake-flask cultures. The platform was proven to be suitable for the screening of a variety of different monoterpenes and offers a useful initial screen to enrich the library with terpene synthase variants that possess the desired properties (e.g. variant product profiles and increased titres) in a single screen, making this method a useful addition to the already existing terpene synthase screening methods that cannot discriminate between different terpene products produced. The pipeline can be easily adapted for the detection of other (mono)terpenoids by adjusting the automated data extraction scripts. The variant product profiles obtained in this study provide a more detailed understanding of the roles of the three plasticity regions in the cyclisation cascade catalysed by PinS. Regions 1 and 3 show a high level of plasticity, where changes in amino acid composition result in active variants with altered product profiles. Amino acids in region 2 are shown to be essential for carbocation isomerisation and/or cyclisation, as mutations in this region result in variants that produce mainly linear products or are inactive. The data generated in this study will be used in future efforts towards the creation of designer terpene synthases for the production of clean monoterpenoid products.

## Materials and Methods

### Bacterial strains and media

All *E*. *coli* strains were routinely grown in Lysogeny Broth (LB) or on LB agar plates including antibiotic supplements as appropriate (carbenicillin, 100 μg mL^−1^; kanamycin, 50 μg mL^−1^). Cloning and plasmid propagation was performed using *E*. *coli* Stellar cells (Clontech). Terpenoids production was performed in phosphate buffered Terrific Broth (TB) using *E*. *coli* DH5α cells (NEB 5α, New England Biolabs).

### Library creation

Separate mutagenic primers encoding the NBT degenerate codons were designed for each target amino acid position, the resulting oligonucleotides were obtained from Integrated DNA Technologies. All oligonucleotides used in this study are shown in Table S3 in the Supplementary Information. The megaprimer PCR method was used to create 16 libraries each containing a NBT codon at a single target position^27^. An asymmetric PCR reaction was performed using a 20-fold excess of PinSMut_Rv over the mutagenic Fw primer to create single stranded mutagenic megaprimers (reverse direction). The megaprimers were purified and used in a second symmetric PCR reaction with PinSMut_Fw to generate the full-length gene libraries. The resulting full-length genes were cloned into the pBb-GPPSmTC/S27 vector, linearised with primer pair Vector_IF_Fw and Vector_IF_Rv, using the In-Fusion HD cloning kit (Takara Bio) according to the manufacturer’s instructions. Correct integration of the NBT codon and cloning was confirmed by standard Sanger sequencing. All plasmids used in this study are shown in Table S4 in the Supplementary Information.

### High-throughput monoterpenoid production

All steps were automated using a Hamilton Star robotics platform fitted with both 8- and 96-head liquid handling capacity with pressure sensing liquid detection allowing efficient and reproducible extraction of the organic phase from bacterial cell cultures. An integrated ClarioStar plate reader allowed online monitoring of cell culture densities prior to induction and harvest^24^. Fresh overnight colonies of cells harbouring a pMVA and a pBb-PinS_var3 plasmid were inoculated in TB medium supplemented with 0.4% (w/v) glucose and appropriate antibiotics in 96-deepwell plates and grown at 37 °C with shaking at 1000 rpm in an infors HP Multitron pro plate incubator. After 6 h (A_600_ ~ 0.6) the cultures were induced by the addition of 50 μM isopropyl β-D-1-thiogalactopyranoside (IPTG) and 25 nM anhydro-tetracycline (aTet) and a 40% (v/v) dodecane organic overlay was added. To prevent evaporation of the organic overlay the 96-deep-well culture plates were sealed post-induction using an ALPS 3000 thermo-sealer. The incubation was then continued for a further 24 h at 30 °C with shaking. At the end of the fermentation the organic overlay was separated by centrifugation (3500 × g, 10 min), transferred to fresh 96-well plates, diluted 1:1 with dodecane containing 0.01% (v/v) *sec*-butylbenzene internal standard, and dried over anhydrous MgSO_4_. After a final centrifugation step, the clarified organic phases were transferred to fresh 96-well plates, sealed and analysed for monoterpene content by GC-MS.

### Automated monoterpenoid detection

High-throughput monoterpenoids analysis in the organic phases was performed as described previously^24^ using an Agilent Technologies 7200 accurate mass Q-TOF mass spectrometer coupled to a 7890B BC and equipped with a PAL RSI 85 autosampler. The sample (1 µl) was injected onto a VF-5MS column (30 m × 250 µm i.d., 0.25 μM film thickness, Agilent Technologies) with an inlet temperature of 280 °C and a split ratio of 100:1. Helium was used as the carrier gas with a flow rate of 1.5 ml/min and a pressure of 16.2 psi. The following oven program was used: 100 °C (1 min hold), ramp to 160 °C at 50 °C/min, and ramp to 325 °C at 120 °C/min (1 min hold). The total runtime per sample was 4.6 minutes. The MS was equipped with an electron impact ion source using 70 eV ionisation and a fixed emission at 35 µA. Mass spectra were collected for the range 35–500 m/z with an acquisition rate of 5 spectra/s and an acquisition time of 200 ms/spectrum. The PAL RSI autosampler permitted the analysis of samples from 96-well plates allowing for high-throughput analysis.

Automated data analysis was conducted as described previously^24,26^. Raw Q-TOF vendor binary files were converted to open source mzXML data format^34^ using ProteoWizard msConvert^35^. Automated monoterpenoid identification was conducted using in-house scripts written in R to automatically extract relevant peak areas. Peak picking was performed at the total ion count (TIC) level in each chromatogram. Peak identification was performed by comparing retention times (RT) and primary ions for each compound with those of authentic standards (See Table S5 in the Supplementary Information). Peaks were integrated and normalized to their corresponding internal standard peak area. A ‘threshold’ signal level was determined based on the average background signal for each of the four compounds targeted in controls samples containing solvent only, which was present on all plates tested.

### Automated sequence analysis of active variants

All clones with at least one peak above threshold level were sent for automated Sanger sequencing using the SUPREMERUN 96 plate sequencing service (GATC Biotech/Eurofins Genomics). The obtained nucleotide sequence files were automatically analysed using custom scripts based on the Samtools^36^ and BCFtools^37^ suite of NGS data analysis libraries. Raw nucleotide sequences were ‘cleaned’ as follows: ‘N’ nucleotides as a result from a poor quality reads, ‘mutations’ (essentially sequencing errors) that either appear outside the expected mutation areas or that did not match the NBT degenerate codon were replaced with the corresponding template nucleotide, and any missing sequence was added to fill short reads. Consequently, all reported mutations were validated against the expected set of mutations based on their position and the encoding of the degenerate codons. Nucleotide sequences were translated using the Biopython library^38^.

### High-resolution product profile analysis

High-resolution monoterpenoid production profiles for selected variants with known mutations were obtained manually using two-phase shake-flask cultures as described previously^5,8^. Briefly, freshly re-streaked colonies were inoculated in 3 ml TB supplemented with 0.4% (w/v) glucose and the appropriate antibiotics in glass screw capped vials, and induced for 24 h at 30 °C with 50 μM IPTG and 25 nM aTet. A 20% *n*-nonane (v/v) organic layer was added to capture the volatile monoterpenoid products. After induction, the nonane overlay was collected, dried over anhydrous MgSO_4_ and mixed at a 1:1 ratio with ethyl acetate containing 0.01% (v/v) *sec*-butylbenzene as internal standard for GC-MS analysis. The samples were injected onto an Agilent Technologies 7890B GC equipped with an Agilent Technologies 5977A MSD. The products were separated on a DB-WAX column (30 m × 0.32 mm i.d., 0.25 μM film thickness, Agilent Technologies). The injector temperature was set at 240 °C with a split ratio of 20:1 (1 μl injection). The carrier gas was helium with a flow rate of 1 ml min^−1^ and a pressure of 5.1 psi. The following oven program was used: 50 °C (1 min hold), ramp to 68 °C at 5 °C min^−1^ (2 min hold), and ramp to 230 °C at 25 °C min^−1^ (2 min hold). The ion source temperature of the mass spectrometer (MS) was set to 230 °C and spectra were recorded from m/z 50 to m/z 250. Compound identification was carried out using authentic standards and comparison to reference spectra in the NIST library of MS spectra and fragmentation patterns as described previously^5^.

### Data analysis

For each variant product profile a Euclidean distance was calculated between the compound distribution observed for a particular variant and that of the starting sequence (VAR3-PinS), where a Euclidean distance of 0 equates to the same product distribution as VAR3-PinS. A plasticity score for a given amino acid position was calculated as being the intersection between the observed amino acid distribution and a perfectly uniform distribution (i.e. if all amino acids were observed with equal frequency). A perfectly uniform distribution would give a plasticity score of 1. All data and the scripts to reproduce this are publicly available at: https://github.com/neilswainston/sbc-leferink.

## Supplementary information

Supp Info

Table s8

Table S9

Table S6

Table S7

## Data Availability

All data generated or analysed during this study are included in this published article and its Supplementary Information available.
