# Supplementary material for: An automated pipeline for the screening of diverse monoterpene synthase libraries
Source: Sci Rep. 2019 Aug 15;9:11936. doi: 10.1038/s41598-019-48452-2 (PMC6695433; doi:10.1038/s41598-019-48452-2)
Supplement: Supplementary file 1 — Supp Info [file 41598_2019_48452_MOESM1_ESM.docx]

**Supplementary Information**

**An automated pipeline for the screening of diverse monoterpene synthase libraries**

Nicole G. H. Leferink, Mark S. Dunstan, Katherine A. Hollywood, Neil Swainston, Andrew Currin, Adrian J. Jervis, Eriko Takano, Nigel S. Scrutton*

Manchester Synthetic Biology Research Centre for Fine and Speciality Chemicals (SYNBIOCHEM), Manchester Institute of Biotechnology and School of Chemistry, University of Manchester, Manchester, United Kingdom

* Corresponding author:

E-mail: nigel.scrutton@manchester.ac.uk; Tel.: + 44 161 306 5152.

**Table of contents**

Figure S1: Schematic representation of the dual-plasmid monoterpenoid production platform. 3

Table S1: Pseudo-mature protein sequences used in this study. 4

Table S2: NBT degenerate codon. 5

Table S3: Oligonucleotides used in this study. 6

Table S4: Plasmids used in this study. 7

Figure S2: GC-QTOF chromatogram of authentic monoterpene standards. 8

Table S5: Retention times and primary ion input data used for automated data extraction. 9

Figure S3: Product profile validation of native PinS and VAR3-PinS. 10

Figure S4: Normalised peak intensities obtained for each variant using the automated screening pipeline. 11

Table S6: Product profiles and total monoterpenoid titres for each variant obtained in this study. 12

Table S7: Codon and amino acid occurrence in active variants. 14

Table S8: Calculated plasticity scores for each targeted position. 15

References 16


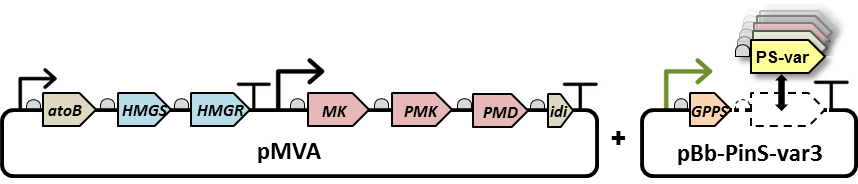


Figure S1: Schematic representation of the dual-plasmid monoterpenoid production platform. pMVA encodes the existing heterologous MVA pathway[^1^](#_ENREF_1) which includes a acetoacetyl-CoA synthase (atoB) and isopentenyl diphosphate isomerase (idi) from *E. coli*, a hydroxymethylglutaryl-CoA synthase (HMGS) and hydroxymethylglutaryl-CoA reductase (HMGR) from *Staphylococcus aureus*, and a mevalonate kinase (MK), phospho-mevalonate kinase (PMK) and phospho-mevalonate decarboxylase (PMD) from *Saccharomyces cerevisiae* under the control of IPTG inducible promoters*.* pBb-PinS-var3 encodes a truncated GPP synthase (GPPS) from *Abies grandis* and a PinS library variant (PS-var) under the control of a tetracycline inducible promoter.

# Table S1: Pseudo-mature protein sequences used in this study.

| **Name** | **Uniprot ID** | **Sequence^a^** |
| --- | --- | --- |
| **(-)aPinS-Pt** | Q84KL6 | MKHHHHHHPMSDYDIPTTENLYFQGAMRRRGDFHSNLWDDDLIQSLSSPYGEP  SYRERAERLIGEVKNSFNSMSNEDGESITPLDDLIQRLWMVDSVERLGIDRHF  KKEIKSALDHVYRYWSEKGIGCGRESVVTDLNSTALGLRTLRLHGYDVSADVL  NHFKNQSGQFACTLKQTEDQIRTVLNLYRASLIAFPGEKVMDEAESFSAKYLK  EALQKIPVSSFSREIGDVLEYGWHTYLPRLEARNYIDVFGQDTENSKSYMKTE  KLLELAKLEFNIFHALQKRELEYLVRWWKGSGSPQMTFCRHRHVEYYTLASCI  AFEPQHSGFRLGFAKACHIITVLDDMYDTFGTLDELELFTSAIKRWDPSATEC  LPEYMKGVYMIVYNTVNEMSQEADKAQGRDTLNYCRQAWEEYIDAYMQEAKWI  ASGEVPTFEEYYENGKVSSGHRVSALQPILTTDIPFPEHVLKEVDIPSQLNDL  ASAILRLRGDTRCYQADRARGEEASCISCYMKDNPGTTEEDALNHLNAMISDV  IKGLNWELLKPNSSVPISAKKHAFDISRAFHCGYKYRDGYSVANIETKSLVKR  TVIDPVTL |
| **VAR3-PinS** | n/a | MKHHHHHHPMSDYDIPTTENLYFQGAMRRRGDFHSNLWDDDLIQSLSSPYGEP  SYRERAERLIGEVKNSFNSMSNEDGESITPLDDLIQRLWMVDSVERLGIDRHF  KKEIKSALDHVYRYWSEKGIGCGRESVVTDLNSTALGLRTLRLHGYDVSADVL  NHFKNQSGQFACTLKQTEDQIRTVLNLYRASLIAFPGEKVMDEAESFSAKYLK  EALQKIPVSSFSREIGDVLEYGWHTYLPRLEARNYIDVFGQDTENSKSYMKTE  KLLELAKLEFNIFHALQKRELEYLVRWWKGSGSPQMTFCRHRHVEYYTLASCI  AFEPQHSGFRLGFAKACHIITVLDDMYDTFGTLDELELFTSAIKRWDPSATEC  LPEYMKGVYMIVYNTVNEMSQEADKAQGRDTLNYCRQAWEEYIDAYMQEAKWI  ASGEVPTFEEYYENGKVSSGHRVSALQPILTTDIPFPEHVLKEVDIPSQLNDL  ASAILRLRGDTRCYQADRARGEEASCISCYMKDNPGTTEEDALNHLNAMISDV  IKGLNWELLKPNSSVPISAKKHAFDIARMAQFMYKYRDGYSVANIETKSLVKR  TVIDPVTL |

^a^ Amino acid sequence of the recombinant proteins as used in this study. The N-terminal sequence originating from the pETM-11 vector, including the TEV cleavable His-tag, is shown in grey, and the three plasticity regions are underlined[^2^](#_ENREF_2).

# Table S2: NBT degenerate codon.

| **N (G, A, T, C)** | **B (G, T, C)** | **T** | **Amino acid** |
| --- | --- | --- | --- |
| G | G | T | Glycine (Gly, G) |
| G | T | T | Valine (Val, V) |
| G | C | T | Alanine (Ala, A) |
| A | G | T | Serine (Ser, S) |
| A | T | T | Isoleucine (Ile, I) |
| A | C | T | Threonine (Thr, T) |
| T | G | T | Cysteine (Cys, C) |
| T | T | T | Phenylalanine (Phe, F) |
| T | C | T | Serine (Ser, S) |
| C | G | T | Arginine (Arg, R) |
| C | T | T | Leucine (Leu, L) |
| C | C | T | Proline (Pro, P) |

Table S3: Oligonucleotides used in this study. The NBT codon is in bold.

| **Name** | **Sequence (5’ → 3’)** |
| --- | --- |
| 335Mut_Fw | GGGCTTTGCAAAAGCA**NBT**CATATTATTACCGTTCTGGATGATATGTAC |
| 336Mut_Fw | GGGCTTTGCAAAAGCATGC**NBT**ATTATTACCGTTCTGGATGATATGTAC |
| 337Mut_Fw | GGGCTTTGCAAAAGCATGCCAT**NBT**ATTACCGTTCTGGATGATATGTAC |
| 338Mut_Fw | GGGCTTTGCAAAAGCATGCCATATT**NBT**ACCGTTCTGGATGATATGTAC |
| 443Mut_Fw | GAGTATTATGAAAATGGTAAAGTTAGC**NBT**GGTCATCGTGTGAGCGCCCTGCAGCC |
| 444Mut_Fw | GAGTATTATGAAAATGGTAAAGTTAGCAGC**NBT**CATCGTGTGAGCGCCCTGCAGCC |
| 445Mut_Fw | GAGTATTATGAAAATGGTAAAGTTAGCAGCGGT**NBT**CGTGTGAGCGCCCTGCAGCC |
| 446Mut_Fw | GAGTATTATGAAAATGGTAAAGTTAGCAGCGGTCAT**NBT**GTGAGCGCCCTGCAGCC |
| 447Mut_Fw | GAGTATTATGAAAATGGTAAAGTTAGCAGCGGTCATCGT**NBT**AGCGCCCTGCAGCC |
| 448Mut_Fw | GAGTATTATGAAAATGGTAAAGTTAGCAGCGGTCATCGTGTG**NBT**GCCCTGCAGCC |
| 557Mut_Fw | GCCAAGAAACATGCATTTGATATT**NBT**CGTATGGCGCAGTTTATG |
| 559Mut_Fw | GCATTTGATATTGCGCGT**NBT**GCGCAGTTTATGTATAAATATCGTGATGGTTACTCTGTTG |
| 560Mut_Fw | GCATTTGATATTGCGCGTATG**NBT**CAGTTTATGTATAAATATCGTGATGGTTACTCTGTTG |
| 561Mut_Fw | GCATTTGATATTGCGCGTATGGCG**NBT**TTTATGTATAAATATCGTGATGGTTACTCTGTTG |
| 562Mut_Fw | GCATTTGATATTGCGCGTATGGCGCAG**NBT**ATGTATAAATATCGTGATGGTTACTCTGTTG |
| 563Mut_Fw | GCATTTGATATTGCGCGTATGGCGCAGTTT**NBT**TATAAATATCGTGATGGTTACTCTGTTG |
| PinSMut_Rv | GTGGTGCTCGAGTTAC |
| PinSMut_Fw | CATCCCCACTACTGAGAATCTTTATTTTCAGGGCG |
| Vector_IF_Fw | TAACTCGAGCACCACCACCACC |
| Vector_IF_Rv | TCAGTAGTGGGGATGTCGTAATCG |

Table S4: Plasmids used in this study. Amino acid numbering is based on the full-length recombinant protein sequence. GenBank files are available online for pGPPSmTC/S27 and pBb-PinS_var3 encoding native PinS and VAR3-PinS respectively.

| **Plasmid reference** | **Plasmid name** | **Description*** | **Source** |
| --- | --- | --- | --- |
| pMVA | BbA5a-MTSAe-T1f-MBI(f)-T1002i | p15A, Kanr, PlacUV5, MTSA, T1, MBI-f, T1002 | [^3^](#_ENREF_3) |
| pGPPSmTC/S27 | pBbB2a-trAgGPPS(co)-(-)aPinS_Pt | pBBR, Ampr, Ptet, trAgGPPS(co)-(-)aPinS_Pt | [^3^](#_ENREF_3) |
| pBb-PinS_var3 | pBbB2a-trAgGPPS(co)- tr(-)aPinS_Pt S557A, A559M, F560A, H561Q, C562F, G563M | pBBR, Ampr, Ptet, trAgGPPS(co)- tr(-)aPinS_Pt S557A, A559M, F560A, H561Q, C562F, G563M | [^4^](#_ENREF_4) |
| pBb-PinS_var3_335m | pBbB2a-trAgGPPS(co)- tr(-)aPinS_Pt C335m, S557A, A559M, F560A, H561Q, C562F, G563M | pBBR, Ampr, Ptet, trAgGPPS(co)- tr(-)aPinS_Pt C335m, S557A, A559M, F560a, H561Q, C562F, G563M | This study |
| pBb-PinS_var3_336m | pBbB2a-trAgGPPS(co)- tr(-)aPinS_Pt H336m, S557A, A559M, F560A, H561Q, C562F, G563M | pBBR, Ampr, Ptet, trAgGPPS(co)- tr(-)aPinS_Pt H336m, S557A, A559M, F560A, H561Q, C562F, G563M | This study |
| pBb-PinS_var3_337m | pBbB2a-trAgGPPS(co)- tr(-)aPinS_Pt I337m, S557A, A559M, F560M, H561Q, C562F, G563M | pBBR, Ampr, Ptet, trAgGPPS(co)- tr(-)aPinS_Pt I337m, S557A, A559M, F560A, H561Q, C562F, G563M | This study |
| pBb-PinS_var3_338m | pBbB2a-trAgGPPS(co)- tr(-)aPinS_Pt I338m, S557A, A559M, F560A, H561Q, C562F, G563M | pBBR, Ampr, Ptet, trAgGPPS(co)- tr(-)aPinS_Pt I338m, S557A, A559M, F560A, H561Q, C562F, G563M | This study |
| pBb-PinS_var3_443m | pBbB2a-trAgGPPS(co)- tr(-)aPinS_Pt S443m, S557A, A559M, F560A, H561Q, C562F, G563M | pBBR, Ampr, Ptet, trAgGPPS(co)- tr(-)aPinS_Pt S443m, S595A, A597M, F598A, H599Q, C600F, G601M | This study |
| pBb-PinS_var3_444m | pBbB2a-trAgGPPS(co)- tr(-)aPinS_Pt G444m, S557A, A559M, F560A, H561Q, C562F, G563M | pBBR, Ampr, Ptet, trAgGPPS(co)- tr(-)aPinS_Pt G444m, S557A, A559M, F560A, H561Q, C562F, G563M | This study |
| pBb-PinS_var3_445m | pBbB2a-trAgGPPS(co)- tr(-)aPinS_Pt H445m, S557A, A559M, F560A, H561Q, C562F, G563M | pBBR, Ampr, Ptet, trAgGPPS(co)- tr(-)aPinS_Pt H445m, S557A, A559M, F560A, H561Q, C562F, G563M | This study |
| pBb-PinS_var3_446m | pBbB2a-trAgGPPS(co)- tr(-)aPinS_Pt R446m, S557A, A559M, F560A, H561Q, C562F, G563M | pBBR, Ampr, Ptet, trAgGPPS(co)- tr(-)aPinS_Pt R446m, S557A, A559M, F560A, H561Q, C562F, G563M | This study |
| pBb-PinS_var3_447m | pBbB2a-trAgGPPS(co)- tr(-)aPinS_Pt V447m, S557A, A559M, F560A, H561Q, C562F, G563M | pBBR, Ampr, Ptet, trAgGPPS(co)- tr(-)aPinS_Pt V447m, S557A, A559M, F560A, H561Q, C562F, G563M | This study |
| pBb-PinS_var3_448m | pBbB2a-trAgGPPS(co)- tr(-)aPinS_Pt S448m, S557A, A559M, F560A, H561Q, C562F, G563M | pBBR, Ampr, Ptet, trAgGPPS(co)- tr(-)aPinS_Pt S448m, S557A, A559M, F560A, H561Q, C562F, G563M | This study |
| pBb-PinS_var3_557m | pBbB2a-trAgGPPS(co)- tr(-)aPinS_Pt S557m, A559M, F560A, H561Q, C562F, G563M | pBBR, Ampr, Ptet, trAgGPPS(co)- tr(-)aPinS_Pt S557m, A559M, F560A, H561Q, C562F, G563M | This study |
| pBb-PinS_var3_559m | pBbB2a-trAgGPPS(co)- tr(-)aPinS_Pt S557A, A559m, F560A, H561Q, C562F, G563M | pBBR, Ampr, Ptet, trAgGPPS(co)- tr(-)aPinS_Pt S557A, A559m, F560A, H561Q, C562F, G563M | This study |
| pBb-PinS_var3_560m | pBbB2a-trAgGPPS(co)- tr(-)aPinS_Pt S557A, A559M, F560m, H561Q, C562F, G563M | pBBR, Ampr, Ptet, trAgGPPS(co)- tr(-)aPinS_Pt S557A, A559M, F560m, H561Q, C562F, G563M | This study |
| pBb-PinS_var3_561m | pBbB2a-trAgGPPS(co)- tr(-)aPinS_Pt_ S557A, A559M, F560A, H561m, C562F, G563M | pBBR, Ampr, Ptet, trAgGPPS(co)- tr(-)aPinS_Pt_ S557A, A559M, F560A, H561m, C562F, G563M | This study |
| pBb-PinS_var3_562m | pBbB2a-trAgGPPS(co)- tr(-)aPinS_Pt_ S557A, A559M, F560A, H561Q, C562m, G563M | pBBR, Ampr, Ptet, trAgGPPS(co)- tr(-)aPinS_Pt_ S557A, A559M, F560A, H561Q, C562m, G563M | This study |
| pBb-PinS_var3_563m | pBbB2a-trAgGPPS(co)- tr(-)aPinS_Pt_ S557A, A559M, F560A, H561Q, C562F, G563m | pBBR, Ampr, Ptet, trAgGPPS(co)- tr(-)aPinS_Pt_ S557A, A559M, F560A, H561Q, C562F, G563m | This study |

*Origin of replication, Antibiotic marker, Reference(s), Promotors and Operons


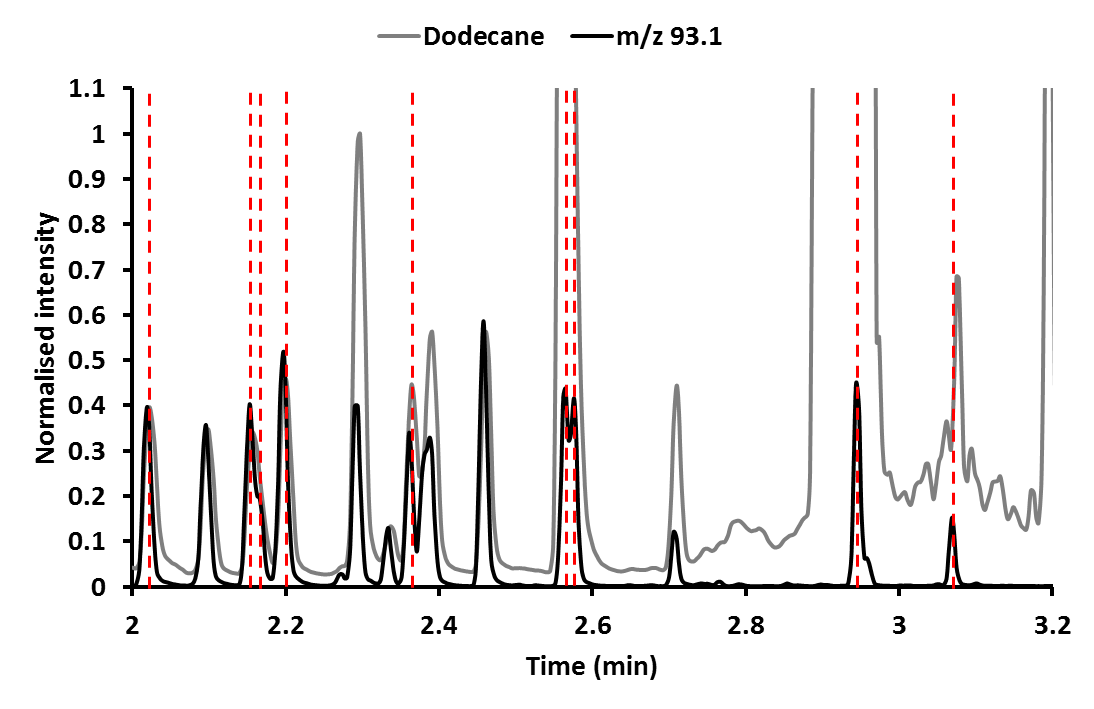


Figure S2: GC-QTOF chromatogram of authentic monoterpene standards. GC-QTOF trace showing the separation of a monoterpenoid mixture (1 mg mL^-1^ each) in ethyl acetate:dodecane (1:1) on a VF-5ms column. The internal standard (IS) used, *sec*-butylbenzene (0.005%, v/v), has a retention time of 2.297 minutes. The standard mixture contained: α-pinene (rt: 2.022), camphene (rt: 2.099), sabinene (rt: 2.153), β-myrcene (rt: 2.166), β-pinene (rt: 2.197), 3-carene (2.297), (*Z*)-β-ocimene (rt: 2.337), limonene (rt: 2.364), (*E*)-β-ocimene (rt: 2.381), 1,8-cineole (rt: 2.388), γ-terpinene (rt: 2.461), terpinolene (rt: 2.565), linalool (rt: 2.576), endo-fenchol (rt: 2.710), α-terpineol (rt: 2.944) and geraniol (rt: 3.069). See materials and methods for details of the method used. The peak intensities were normalised against the peak intensity of *sec*-butylbenzene. The Total Ion Chromatogram (TIC) is shown in grey and the Extracted Ion Chromatogram (EIC) of m/z 93.1, a prominent ion for all monoterpenoid peaks, is shown in black. The eight peaks relevant for VAR3-PinS are marked with red dashed lines.

#

# Table S5: Retention times and primary ion input data used for automated data extraction.

| **Name** | **RT (min)** | **Primary Ion (m/z)** |
| --- | --- | --- |
| α-pinene | 2.019 | 93.1 |
| β-pinene | 2.197 | 93.1 |
| Sabinene | 2.153 | 93.1 |
| β-myrcene | 2.166 | 93.1 |
| Limonene | 2.361 | 67.1 |
| Terpinolene | 2.565 | 93.1 |
| Linalool | 2.576 | 93.1 |
| α-terpineol | 2.944 | 121.1 |
| Geraniol | 3.069 | 69.1 |


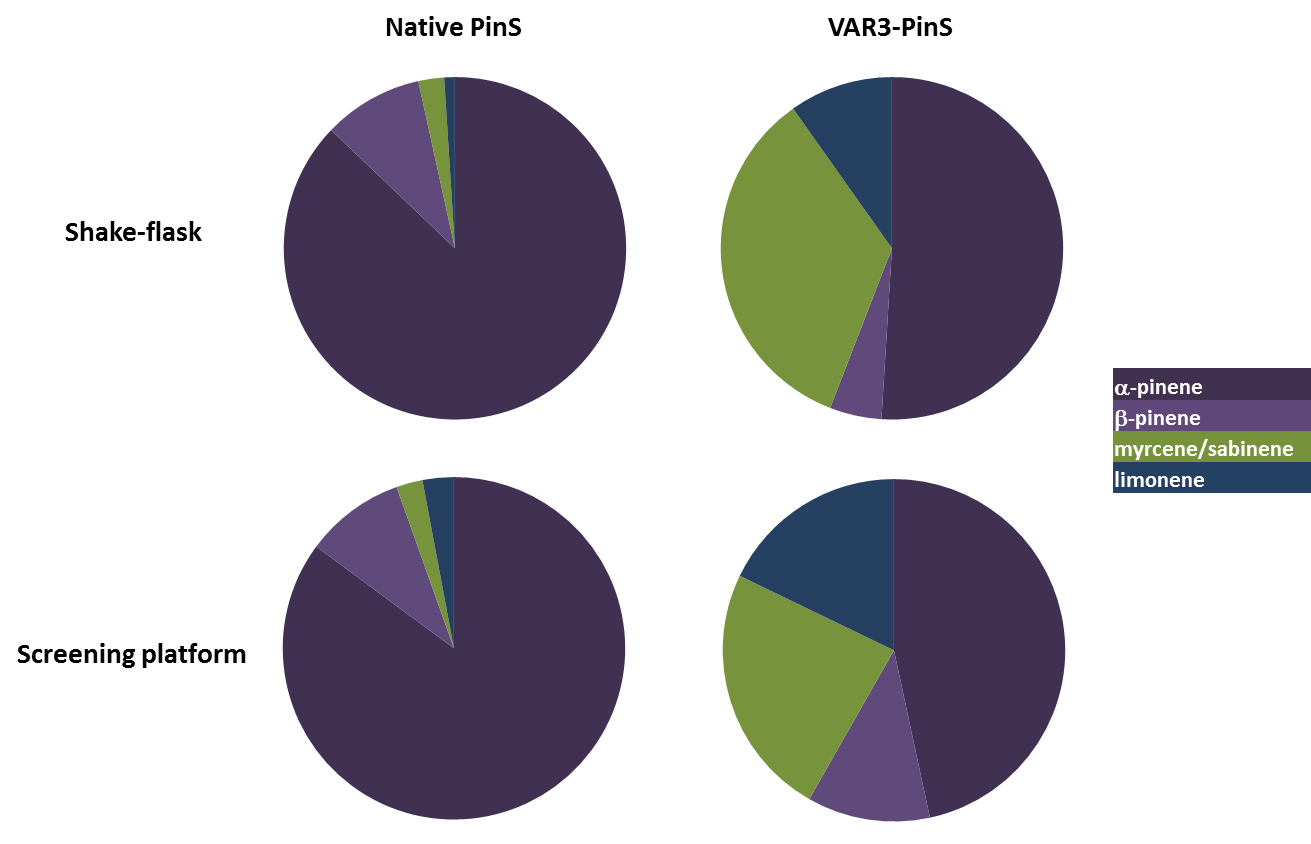


Figure S3: Product profile validation of native PinS and VAR3-PinS. Comparison of relative product profiles of native PinS and VAR3-PinS upon targeted analysis of four key product peaks obtained using the automated pipeline compared to the same products obtained in conventional shake-flask cultures. The number of colonies screened using the pipeline was 45 and 57 for native PinS and VAR3-PinS respectively. The shake-flask data was obtained from Leferink *et al* 2018[^4^](#_ENREF_4).

**
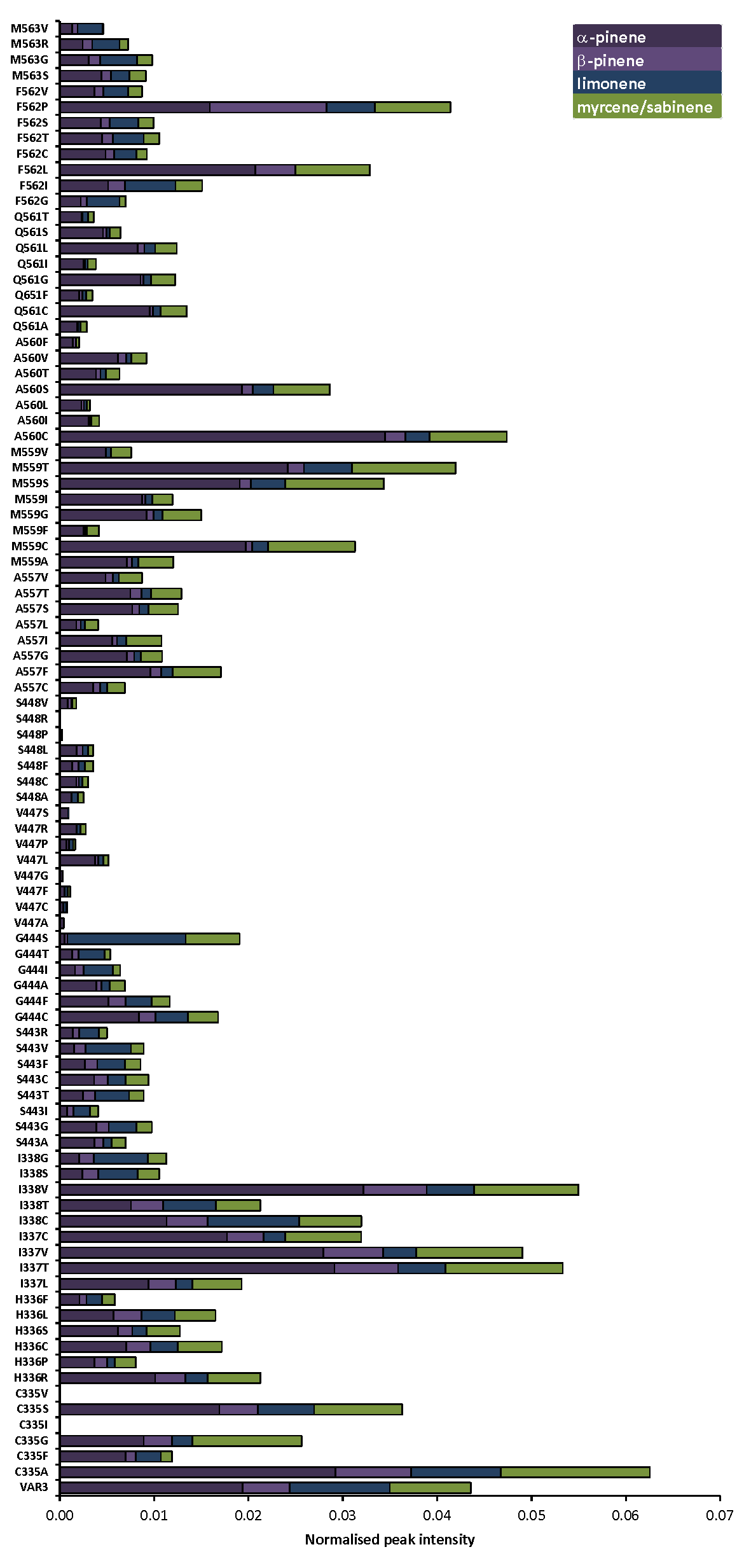
**

Figure S4: Normalised peak intensities obtained for each variant using the automated screening pipeline. Peaks were integrated and normalized to their corresponding internal standard peak area and corrected for the background signal.

Table S6: Product profiles and total monoterpenoid titres for each variant obtained in this study. Product profiles and monoterpenoid titres (mg L_org_^-1^) are determined from two-phase cultures with an overlay for each re-streaked *E. coli* strain containing the MVA pathway and a unique variant PinS. The main product for each strain is shown in bold. Data for native PinS and VAR3-PinS are obtained from Leferink *et al* 2018[^4^](#_ENREF_4). This table is also available as spreadsheet.

|  | **Monoterpenoids** | | | | | | | | | | | | **Sesquiterpenoids** | | |
| --- | --- | --- | --- | --- | --- | --- | --- | --- | --- | --- | --- | --- | --- | --- | --- |
| **PinS** | **geraniol** | **myrcene** | **linalool** | **limonene** | **α-terpineol** | **β-phellandrene** | **terpinolene** | **α-pinene** | **β-pinene** | **sabinene** | **sabinene-hydrate** | **Other^a^** | **farnesene** | **nerolidol** | **farnesol** |
| Native | 18.3 | 20.3 |  | 10.5 | 1.9 | 11.2 | 0.8 | 749.8 | 103.1 | 0.5 | 0.5 | 11.3 |  |  |  |
| VAR3 | 25.1 | 2.0 | 0.9 | 1.0 | 3.5 |  | 0.4 | 5.2 | 0.5 | 1.5 |  |  |  |  |  |
| [C335A] | 41.7 | 3.0 | 0.8 | 2.5 | 4.3 | 0.6 | 0.7 | 9.4 |  | 3.9 | 1.1 |  |  |  |  |
| [C335F] | 12.6 |  |  |  |  |  |  |  |  |  |  |  |  |  |  |
| [C335G] | 72.3 | 5.3 | 1.0 | 2.1 | 5.1 | 0.7 | 13.1 | 7.3 | 0.3 | 21.4 | 6.0 |  |  |  |  |
| [C335I] | 15.4 |  |  |  |  |  |  |  |  |  |  |  |  |  |  |
| [C335S] | 29.2 | 2.6 | 0.7 | 2.3 | 10.8 |  | 0.4 | 6.2 | 0.5 | 2.1 | 0.5 |  |  |  |  |
| [C335V] | 9.2 | 0.2 | 0.1 |  | 0.1 |  |  |  |  |  |  |  |  |  |  |
| [H336P] | 6.8 |  |  |  |  |  |  |  |  |  |  |  |  |  |  |
| [I337L] | 41.8 | 0.3 |  |  | 0.7 |  |  | 1.3 |  |  |  |  |  |  |  |
| [I337T] | 43.6 | 3.7 | 0.8 | 0.5 | 6.3 |  |  | 9.3 |  | 2.5 |  |  |  |  |  |
| [I337V] | 1.6 |  |  |  | 1.1 |  |  | 1.6 |  |  |  |  |  |  |  |
| [I338C] | 9.4 |  |  |  | 0.6 |  |  |  |  |  |  |  |  |  |  |
| [I338T] | 38.7 | 0.2 |  | 0.2 | 1.9 |  |  | 0.7 |  | 0.3 |  |  |  |  |  |
| [I338V] | 13.9 | 0.5 | 0.4 |  | 2.5 |  |  | 3.0 |  | 0.6 |  |  |  |  |  |
| [S443A] | 41.8 | 2.2 | 1.1 | 0.1 | 4.3 |  |  | 5.3 |  | 0.7 |  |  |  |  |  |
| [S443G] | 136.6 | 2.7 | 1.2 |  | 2.3 |  |  | 2.4 |  | 0.2 |  |  |  |  |  |
| [S443I] | 3.8 |  |  |  |  |  |  |  |  |  |  |  |  |  |  |
| [S443T] | 1.3 |  |  |  |  |  |  |  |  |  |  |  |  |  |  |
| [G444C] | 22.2 |  |  |  |  |  |  |  |  |  |  |  |  |  | 16.8 |
| [V447A] | 81.9 |  |  |  | 0.5 |  |  | 0.5 |  |  |  |  |  |  |  |
| [V447C] | 31.1 |  | 0.3 |  | 2.3 |  |  | 2.9 |  |  |  |  |  |  |  |
| [V447F] | 20.0 |  |  |  |  |  |  |  |  |  |  |  |  |  |  |
| [V447G] | 38.0 |  |  |  |  |  |  |  |  |  |  |  |  |  |  |
| [V447L] | 102.1 | 1.3 | 0.6 |  | 4.4 |  |  | 4.1 |  | 0.5 |  |  |  |  |  |
| [V447P] | 42.4 |  | 0.3 |  | 3.9 |  |  | 3.8 |  | 0.8 |  |  |  |  |  |
| [V447R] | 4.8 |  |  |  |  |  |  |  |  |  |  |  |  |  |  |
| [V447S] | 14.2 |  |  |  |  |  |  |  |  |  |  |  |  |  |  |
| [S448A] | 3.9 |  |  |  |  |  |  |  |  |  |  |  |  |  |  |
| [S448C] | 59.3 |  |  |  | 1.9 |  |  | 5.3 |  |  |  |  |  |  |  |
| [S448F] | 68.8 |  |  |  |  |  |  |  |  |  |  |  |  |  |  |
| [S448L] | 7.1 |  |  |  |  |  |  |  |  |  |  |  |  |  |  |
| [S448P] | 64.6 |  |  |  |  |  |  |  |  |  |  |  |  |  |  |

^a^ Other products detected for native PinS are: camphene, camphene-hydrate, borneol and pinan-2-ol.

**Table S6: Continued.**

|  | **Monoterpenoids** | | | | | | | | | | | | **Sesquiterpenoids** | | |
| --- | --- | --- | --- | --- | --- | --- | --- | --- | --- | --- | --- | --- | --- | --- | --- |
| **PinS** | **geraniol** | **myrcene** | **linalool** | **limonene** | **α-terpineol** | **β-phellandrene** | **terpinolene** | **α-pinene** | **β-pinene** | **sabinene** | **sabinene-hydrate** | **Other** | **farnesene** | **nerolidol** | **farnesol** |
| [S448R] | 130.4 |  |  |  |  |  |  |  |  |  |  |  |  |  |  |
| [S448V] | 29.8 |  |  |  |  |  |  |  |  |  |  |  |  |  |  |
| [A557C] | 9.4 | 0.9 |  | 0.2 | 4.1 |  |  | 3.9 |  | 1.1 |  |  |  |  |  |
| [A557F] | 49.8 |  | 0.3 |  | 3.8 |  |  | 2.0 |  | 1.4 |  |  |  |  |  |
| [A557G] | 30.4 | 1.7 |  |  | 2.5 |  |  | 2.7 |  | 0.4 |  |  |  |  |  |
| [A557I] | 37.8 |  |  | 0.5 | 5.1 |  |  | 3.5 |  | 2.5 |  |  |  |  |  |
| [A557L] | 42.7 |  |  |  | 0.6 |  |  | 0.5 |  |  |  |  |  |  |  |
| [A557S] | 61.6 | 6.5 | 2.0 | 1.4 | 19.4 | 0.9 | 0.3 | 19.4 | 0.4 | 6.6 |  |  |  |  |  |
| [A557T] | 24.5 | 1.7 | 0.6 | 0.3 | 7.0 |  |  | 6.2 |  | 2.1 |  |  |  |  |  |
| [A557V] | 72.1 | 1.3 | 0.2 | 0.2 | 5.3 |  |  | 2.7 |  | 2.0 |  |  |  |  |  |
| [M559A] | 38.0 | 1.5 | 0.3 |  | 3.5 |  |  | 3.1 |  | 1.0 |  |  |  |  |  |
| [M559C] | 22.5 | 6.0 | 0.7 | 0.7 | 10.8 |  |  | 10.3 |  | 3.6 |  |  |  |  |  |
| [M559F] | 57.2 | 0.4 | 4.4 |  | 1.4 |  |  | 0.8 |  |  |  |  |  |  |  |
| [M559G] | 63.1 | 4.7 | 2.9 | 0.9 | 13.5 |  |  | 10.3 |  | 2.5 |  |  |  |  |  |
| [M559I] | 81.0 | 4.8 | 2.0 | 0.7 | 14.1 |  |  | 21.6 | 0.3 | 3.4 |  |  |  |  |  |
| [M559S] | 18.3 | 9.8 | 7.9 | 5.0 | 30.9 |  | 0.4 | 14.8 | 0.2 | 5.0 |  |  |  |  |  |
| [M559T] | 3.3 | 2.1 | 0.5 |  | 2.7 |  |  | 1.3 |  | 0.3 |  |  |  |  |  |
| [M559V] | 38.2 | 11.2 | 0.6 | 2.4 | 26.1 | 0.7 | 0.3 | 21.0 | 0.3 | 6.4 |  |  | 0.8 | 2.0 |  |
| [A560C] | 25.9 | 8.9 | 2.2 | 3.6 | 30.1 |  | 0.9 | 40.2 | 0.9 | 7.3 | 1.1 |  |  |  |  |
| [A560I] | 32.7 |  |  |  | 0.4 |  |  | 0.4 |  |  |  |  |  |  |  |
| [A560L] | 45.4 | 0.5 |  |  |  |  |  |  |  |  |  |  |  |  |  |
| [A560S] | 24.4 | 3.6 | 1.2 | 0.6 | 3.1 |  |  | 5.4 |  | 2.0 |  |  |  |  |  |
| [A560T] | 22.8 | 2.9 | 1.2 | 0.6 | 2.8 |  |  | 5.1 |  | 1.9 |  |  |  |  |  |
| [A560V] | 32.7 |  |  |  | 0.4 |  |  | 0.4 |  |  |  |  |  |  |  |
| [Q561A] | 39.2 | 1.2 | 0.4 | 0.1 | 2.7 |  |  | 2.8 |  | 0.6 |  |  |  |  |  |
| [Q561C] | 46.0 | 7.5 | 2.3 | 1.8 | 7.2 |  | 0.5 | 14.3 | 0.7 | 4.4 |  |  |  |  |  |
| [Q561F] | 63.8 | 1.3 | 0.4 |  | 0.9 |  |  | 0.6 |  |  |  |  | 0.8 |  |  |
| [Q561G] | 32.5 | 2.2 | 1.3 | 0.4 | 2.4 |  |  | 4.3 |  | 0.8 |  |  |  |  |  |
| [Q561I] | 34.4 | 1.9 | 0.6 | 0.5 | 2.0 |  |  | 3.2 |  | 1.5 |  |  |  |  |  |
| [Q561L] | 40.1 | 4.8 | 1.6 | 1.0 | 4.8 |  | 0.4 | 8.7 | 0.4 | 3.4 |  |  |  |  |  |
| [Q561S] | 43.9 | 3.1 | 2.3 | 0.5 | 2.6 |  |  | 5.0 |  | 0.9 |  |  |  |  |  |
| [Q561T] | 26.8 |  |  |  | 0.4 |  |  | 0.6 |  |  |  |  |  |  |  |
| [F562G] | 13.9 |  |  |  |  |  |  |  |  |  |  |  |  |  |  |
| [F562L] | 1.3 |  |  |  | 0.2 |  |  | 0.4 |  |  |  |  |  |  |  |
| [F562I] | 9.0 |  |  |  |  |  |  |  |  |  |  |  |  |  |  |
| [M563S] | 1.4 |  |  |  |  |  |  |  |  |  |  |  |  |  |  |

Table S7: Codon and amino acid occurrence in active variants. The codon and amino acid occurrence at each position in active variants detected using the automated screening pipeline. The most occurring codon for each position is highlighted in bold. This table is also available as spreadsheet.

|  | **Region 1** | | | | **Region 2** | | | | | | **Region 3** | | | | | |  |  |
| --- | --- | --- | --- | --- | --- | --- | --- | --- | --- | --- | --- | --- | --- | --- | --- | --- | --- | --- |
| **Codon** | **335** | **336** | **337** | **338** | **443** | **444** | **445** | **446** | **447** | **448** | **557** | **559** | **560** | **561** | **562** | **563** | **Total** | **Rel. (%)** |
| **ACT** (Thr) | 0 | 0 | 2 | 2 | 3 | 0 | 0 | 0 | 0 | 0 | 3 | 4 | 1 | 1 | 0 | 0 | 16 | 6.0 |
| **CCT** (Pro) | 0 | **4** | 0 | 0 | 0 | 0 | 0 | 0 | 1 | 1 | 0 | 0 | 0 | 0 | 0 | 0 | 6 | 2.2 |
| **GCT** (Ala) | 5 | 0 | 0 | 0 | 4 | 0 | 0 | 0 | 1 | 1 | 4 | 1 | 4 | 1 | 0 | 0 | 21 | 7.9 |
| **TCT** (Ser) | 4 | 0 | 0 | 0 | **5** | 0 | 0 | 0 | 1 | 0 | 2 | 2 | 2 | 2 | 0 | 0 | 18 | 6.8 |
| **AGT** (Ser) | 4 | 0 | 0 | 0 | 3 | 0 | 0 | 0 | 0 | **2** | 2 | 3 | 0 | **5** | 0 | **5** | 24 | 9.1 |
| **CGT** (Arg) | 0 | 0 | 0 | 0 | 0 | 0 | 0 | 0 | 1 | 1 | 0 | 0 | 0 | 0 | 0 | 0 | 2 | 0.8 |
| **GGT** (Gly) | 5 | 0 | 0 | 0 | 4 | **17** | 0 | 0 | **2** | 0 | 2 | 4 | 0 | 2 | 1 | 0 | 37 | 14.0 |
| **TGT** (Cys) | **18** | 0 | 0 | **10** | 0 | 2 | 0 | 0 | 1 | **2** | 2 | **5** | 2 | 5 | 0 | 0 | 47 | 17.4 |
| **ATT** (Ile) | 3 | 0 | 2 | 0 | 1 | 0 | 0 | 0 | 0 | 0 | 1 | **5** | 2 | 3 | 8 | 0 | 25 | 9.4 |
| **CTT** (Leu) | 0 | 0 | 1 | 0 | 0 | 0 | 0 | 0 | 1 | 1 | 2 | 0 | 1 | 5 | 6 | 0 | 17 | 6.4 |
| **GTT** (Val) | 3 | 0 | **7** | 5 | 0 | 0 | 0 | 0 | 1 | 1 | 3 | 1 | 2 | 0 | 0 | 0 | 23 | 8.7 |
| **TTT** (Phe) | 4 | 0 | 0 | 0 | 0 | 0 | 0 | 0 | 1 | **2** | **6** | 2 | 0 | 3 | **11** | 0 | 29 | 10.9 |

Table S8: Calculated plasticity scores for each targeted position. The plasticity score for each amino acid position was calculated as being the intersection between the observed amino acid distribution and a perfectly uniform distribution. This table is also available as spreadsheet.

| **Position** | **WT residue** | **VAR3 residue** | **plasticity** |
| --- | --- | --- | --- |
| 335 | C | C | 0.581028 |
| 336 | H | H | 0.090909 |
| 337 | I | I | 0.356061 |
| 338 | I | I | 0.272727 |
| 443 | S | S | 0.413636 |
| 444 | G | G | 0.181818 |
| 445 | H | H | 0 |
| 446 | R | R | 0 |
| 447 | V | V | 0.818182 |
| 448 | S | S | 0.727273 |
| 557 | S | A | 0.713805 |
| 559 | A | M | 0.602694 |
| 560 | F | A | 0.597403 |
| 561 | H | Q | 0.602694 |
| 562 | C | F | 0.311189 |
| 563 | G | M | 0.090909 |

# References

1 Alonso-Gutierrez, J. *et al.* Metabolic engineering of *Escherichia coli* for limonene and perillyl alcohol production. *Metab. Eng.* **19**, 33-41 (2013).

2 Leferink, N. G. H. *et al.* Experiment and simulation reveal how mutations in functional plasticity regions guide plant monoterpene synthase product outcome. *ACS Catalysis* **8**, 3780-3791 (2018).

3 Leferink, N. G. H. *et al.* A ‘plug and play’ platform for the production of diverse monoterpene hydrocarbon scaffolds in *Escherichia coli*. *ChemistrySelect* **1**, 1893-1896 (2016).

4 Leferink, N. G. H. *et al.* Experiment and simulation reveal how mutations in functional plasticity regions guide plant monoterpene synthase product outcome. *ACS Catal.* **8**, 3780-3791 (2018).
